# Supplementary material for: The use of early warning system scores in prehospital and emergency department settings to predict clinical deterioration: A systematic review and meta-analysis
Source: PLoS One. 2022 Mar 17;17(3):e0265559. doi: 10.1371/journal.pone.0265559 (PMC8929648; doi:10.1371/journal.pone.0265559)
Supplement: S1 File — (PDF) [file pone.0265559.s002.pdf]

Supplementary file 1 PICO question

Population

Adult population who presented to Emergency Departments (EDs).

Interventions

All 5 EWS scores will be interventions for the target population.

1. Modified Early Warning Scores
2. National Early Warning Scores
3. National Early Warning Scores 2
4. Rapid Acute Physiological Score
5. Cardiac Arrest Risk Triage

Comparison

Nil

Outcomes

- Mortality rate (3 and 30 days)
- Length of stay
- Cardiac arrest
- Respiratory arrest
- ICU admission
