# Supplementary material for: The use of early warning system scores in prehospital and emergency department settings to predict clinical deterioration: A systematic review and meta-analysis
Source: PLoS One. 2022 Mar 17;17(3):e0265559. doi: 10.1371/journal.pone.0265559 (PMC8929648; doi:10.1371/journal.pone.0265559)
Supplement: S2 File — (PDF) [file pone.0265559.s003.pdf]

Supplementary file 2 **Detailed search history**

EMBASE SEARCH HISTORY

Ovid Technologies, Inc. Email Service

Search for: limit 15 to yr="2020 -Current"

Results: 290

Database: Embase Classic+Embase <1947 to 2021 February 15>
Search Strategy:

----

1 exp *Emergency Medical Technicians/ or exp *Ambulances/ (8562)

2 (paramedic* or ems or emergency medical service* or pre-hospital or pre-hospital or ambulance* or emergency medical
technician* or emt or (out-of-hospital or "out of hospital")).mp. [mp=title, abstract, heading word, drug trade name,
original title, device manufacturer, drug manufacturer, device trade name, keyword, floating subheading word, candidate
term word] (136461)

3 exp *Emergency Service, Hospital/ (1488)

4 (emergency department* or emergency room* or "accident* and emergency" or "accident* & emergency" or "a&e" or "a &
e" or "A and E" or ("ER" or " ED")).mp. [mp=title, abstract, heading word, drug trade name, original title, device
manufacturer, drug manufacturer, device trade name, keyword, floating subheading word, candidate term word] (1808541)

5 exp *"severity of illness index"/ or exp *early warning score/ (2042)

6 (early warning sign* or early warning system* or early warning score* or early warning

score system* or ews or
trigger score or ("track and trigger" or "track and trigger system*")).mp. [mp=title, abstract, heading word, drug trade
name, original title, device manufacturer, drug manufacturer, device trade name, keyword, floating subheading word,
candidate term word] (9483)

7 (national early warning score2* or national early warning score system 2* or NEWS2 or NEWS 2).mp. [mp=title,
abstract, heading word, drug trade name, original title, device manufacturer, drug manufacturer, device trade name,
keyword, floating subheading word, candidate term word] (160)

8 (national early warning score* or national early warning score system* or NEWS).mp. [mp=title, abstract, heading
word, drug trade name, original title, device manufacturer, drug manufacturer, device trade name, keyword, floating
subheading word, candidate term word] (26024)

9 (modified early warning score* or modified early warning score system* or MEWS).mp. [mp=title, abstract, heading
word, drug trade name, original title, device manufacturer, drug manufacturer, device trade name, keyword, floating
subheading word, candidate term word] (707)

10 (rapid acute physiology score* or rapid acute physiology score system* or RAPS).mp. [mp=title, abstract, heading
word, drug trade name, original title, device manufacturer, drug manufacturer, device trade name, keyword, floating
subheading word, candidate term word] (543)

11 (Cardiac arrest risk triage or Cardiac arrest risk triage score or CART).mp. [mp=title, abstract, heading word,
drug trade name, original title, device manufacturer, drug manufacturer, device trade name, keyword, floating subheading
word, candidate term word] (13244)

12 1 or 2 or 3 or 4 (1930574)

13 5 or 6 or 7 or 8 or 9 or 10 or 11 (50499)

14 12 and 13 (3433)

15 limit 14 to english language (3145)

16 limit 15 to yr="2020 -Current" (290)

MEDLINE SEARCH HISTORY

Ovid Technologies, Inc. Email Service

Search for: limit 15 to yr="2020 -Current"

Results: 253

Database: Ovid MEDLINE(R) ALL <1946 to February 15, 2021>

Search Strategy:

----

1 exp *Emergency Medical Technicians/ or exp *Ambulances/ (9735)

2 (paramedic* or ems or emergency medical service* or pre-hospital or pre-hospital or ambulance* or emergency medical technician* or emt or (out-of-hospital or "out of hospital")).mp. [mp=title, abstract, original title, name of substance word, subject heading word, floating sub-heading word, keyword heading word, organism supplementary concept word, protocol supplementary concept word, rare disease supplementary concept word, unique identifier, synonyms] (111027)

3 exp *Emergency Service, Hospital/ (47656)

4 (emergency department* or emergency room* or "accident* and emergency" or "accident* & emergency" or "a&e" or "a & e" or "A and E" or ("ER" or " ED")).mp. [mp=title, abstract, original title, name of substance word, subject heading word, floating sub-heading word, keyword heading word, organism supplementary concept word, protocol supplementary concept word, rare disease supplementary concept word, unique identifier, synonyms] (315256)

5 exp *"severity of illness index"/ or exp *early warning score/ (22344)

6 (early warning sign* or early warning system* or early warning score* or early warning

score system* or ews or
trigger score or ("track and trigger" or "track and trigger system*")).mp. [mp=title, abstract, original title, name of
substance word, subject heading word, floating sub-heading word, keyword heading word, organism supplementary concept
word, protocol supplementary concept word, rare disease supplementary concept word, unique identifier, synonyms] (6582)
7 (national early warning score2* or national early warning score system 2* or NEWS2 or NEWS 2).mp. [mp=title,
abstract, original title, name of substance word, subject heading word, floating sub-heading word, keyword heading word,
organism supplementary concept word, protocol supplementary concept word, rare disease supplementary concept word,
unique identifier, synonyms] (107)
8 (national early warning score* or national early warning score system* or NEWS).mp. [mp=title, abstract, original
title, name of substance word, subject heading word, floating sub-heading word, keyword heading word, organism
supplementary concept word, protocol supplementary concept word, rare disease supplementary concept word, unique
identifier, synonyms] (225325)
9 (modified early warning score* or modified early warning score system* or MEWS).mp. [mp=title, abstract, original
title, name of substance word, subject heading word, floating sub-heading word, keyword heading word, organism
supplementary concept word, protocol supplementary concept word, rare disease supplementary concept word, unique
identifier, synonyms] (396)
10 (rapid acute physiology score* or rapid acute physiology score system* or RAPS).mp. [mp=title, abstract, original
title, name of substance word, subject heading word, floating sub-heading word, keyword heading word, organism

supplementary concept word, protocol supplementary concept word, rare disease supplementary concept word, unique
identifier, synonyms] (370)

11 (Cardiac arrest risk triage or Cardiac arrest risk triage score or CART).mp. [mp=title, abstract, original title,
name of substance word, subject heading word, floating sub-heading word, keyword heading word, organism supplementary
concept word, protocol supplementary concept word, rare disease supplementary concept word, unique identifier, synonyms]
(8105)

12 1 or 2 or 3 or 4 (427803)

13 5 or 6 or 7 or 8 or 9 or 10 or 11 (261999)

14 12 and 13 (3575)

15 limit 14 to english language (3347)

16 limit 15 to yr="2020 -Current" (253)

CINAHL search history

S13 cardiac arrest risk triage or cardiac arrest risk triage score OR Expanders - Apply equivalent subjects Interface - EBSCOhost Research Databases 2,617

CART Search modes - Boolean/Phrase Search Screen - Advanced Search

Database - CINAHL

S12 S9 AND S10 Limiters - Published Date: 20200101-20211231 Interface - EBSCOhost Research Databases 166

Expanders - Apply equivalent subjects Search Screen - Advanced Search

Search modes - Boolean/Phrase Database - CINAHL

S11 S9 AND S10 Expanders - Apply equivalent subjects Interface - EBSCOhost Research Databases 2,432

Search modes - Boolean/Phrase Search Screen - Advanced Search

Database - CINAHL

S10 S3 OR S4 OR S5 OR S6 OR S7 OR S8 Expanders - Apply equivalent subjects Interface - EBSCOhost Research Databases 75,896

Search modes - Boolean/Phrase Search Screen - Advanced Search

Database - CINAHL

S9 S1 OR S2 Expanders - Apply equivalent subjects Interface - EBSCOhost Research Databases 325,316

Search modes - Boolean/Phrase Search Screen - Advanced Search

Database - CINAHL

S8 cardiac arrest risk triage or cardiac arrest risk triage score OR Expanders - Apply equivalent subjects Interface - EBSCOhost Research Databases 2,617

CART Search modes - Boolean/Phrase Search Screen - Advanced Search

Database - CINAHL

S7 rapid acute physiology score* or rapid acute physiology score Expanders - Apply equivalent subjects Interface - EBSCOhost Research Databases 866

system or RAPS Search modes - Boolean/Phrase Search Screen - Advanced Search

Database - CINAHL

S6 modified early warning score* or modified early warning score Expanders - Apply equivalent subjects Interface - EBSCOhost Research Databases 276

system* or MEWS Search modes - Boolean/Phrase Search Screen - Advanced Search

Database - CINAHL

S5 national early warning score 2 or national early warning score2 Expanders - Apply equivalent subjects Interface - EBSCOhost Research Databases 72

or NEWS2 or "NEWS 2" Search modes - Boolean/Phrase Search Screen - Advanced Search

Database - CINAHL

S4 national early warning score* or national early warning score Expanders - Apply equivalent subjects Interface - EBSCOhost Research Databases 70,536

system* or NEWS Search modes - Boolean/Phrase Search Screen - Advanced Search

Database - CINAHL

S3 early warning sign* or early warning system* or early warning Expanders - Apply equivalent subjects Interface - EBSCOhost Research Databases 2,132

score* or early warning score or ews or trigger score or "track Search modes - Boolean/Phrase Search Screen - Advanced Search

and trigger" or "track and trigger system" Database - CINAHL

S2 emergency department* or emergency room* or "accident* Expanders - Apply equivalent subjects Interface - EBSCOhost Research Databases 205,895

and emergency" or "accident* & emergency" or "a&e" or "a & Search modes - Boolean/Phrase Search Screen - Advanced Search

e" or "A and E" or "ER" or "ED" Database - CINAHL

S1 paramedic* or ems or emergency medical service* or Expanders - Apply equivalent subjects Interface - EBSCOhost Research Databases 130,491

prehospital or pre-hospital or ambulance* or emergency Search modes - Boolean/Phrase Search Screen - Advanced Search

medical technician* or met OR out-of-hospital or "out of hospital”

Web of Science Search History

13 362

#10 AND #9

Refined by: LANGUAGES: (ENGLISH) AND PUBLICATION YEARS: (2021 OR 2020)

Databases= WOS, CCC, KJD, MEDLINE, RSCI, SCIELO Timespan=All years Search language=Auto

12 2,638

#10 AND #9

Refined by: LANGUAGES: (ENGLISH)

Databases= WOS, CCC, KJD, MEDLINE, RSCI, SCIELO Timespan=All years Search language=Auto

11 2,860

#10 AND #9

Databases= WOS, CCC, KJD, MEDLINE, RSCI, SCIELO Timespan=All years Search language=Auto

10 198,920

#8 OR #7 OR #6 OR #5 OR #4 OR #3

Databases= WOS, CCC, KJD, MEDLINE, RSCI, SCIELO Timespan=All years Search language=Auto

9 692,366

#2 OR #1

Databases= WOS, CCC, KJD, MEDLINE, RSCI, SCIELO Timespan=All years Search language=Auto

8 21,363

TOPIC: (Cardiac arrest risk triage or Cardiac arrest risk triage score or CART)

Databases= WOS, CCC, KJD, MEDLINE, RSCI, SCIELO Timespan=All years Search language=Auto

7 15,559

TOPIC: (rapid acute physiology score* or rapid acute physiology score system* or RAPS)

Databases= WOS, CCC, KJD, MEDLINE, RSCI, SCIELO Timespan=All years Search language=Auto

6 2,095

TOPIC: (modified early warning score* or modified early warning score system* or MEWS)

Databases= WOS, CCC, KJD, MEDLINE, RSCI, SCIELO Timespan=All years Search language=Auto

5 123,039 TOPIC: (national early warning score* or national early warning score system* or NEWS)

Databases= WOS, CCC, KJD, MEDLINE, RSCI, SCIELO Timespan=All years Search language=Auto

4 11,072

TOPIC: (national early warning score 2 or national early warning score system 2 or NEWS2 or NEWS 2)

Databases= WOS, CCC, KJD, MEDLINE, RSCI, SCIELO Timespan=All years Search language=Auto

3 38,428

TOPIC:(early warning sign* or early warning system* or early warning score* or early warning score system* or ews or trigger score or "track and trigger" or "track and trigger system*")

Databases= WOS, CCC, KJD, MEDLINE, RSCI, SCIELO Timespan=All years Search language=Auto

2 543,490

TOPIC:(emergency department* or emergency room* or "accident* and emergency" or "accident* & emergency" or "a&e" or "a & e" or "A and E" or "ER" or " ED")

Databases= WOS, CCC, KJD, MEDLINE, RSCI, SCIELO Timespan=All years Search language=Auto

1 199,218

TOPIC:(paramedic* or ems or emergency medical service* or prehospital or pre-hospital or ambulance* or emergency medical technician* or emt or out-of- hospital or "out of hospital")

Databases= WOS, CCC, KJD, MEDLINE, RSCI, SCIELO Timespan=All years Search language=Auto
