# Supplementary material for: The use of early warning system scores in prehospital and emergency department settings to predict clinical deterioration: A systematic review and meta-analysis
Source: PLoS One. 2022 Mar 17;17(3):e0265559. doi: 10.1371/journal.pone.0265559 (PMC8929648; doi:10.1371/journal.pone.0265559)
Supplement: S3 File — (PDF) [file pone.0265559.s004.pdf]

Supplementary file 3 Risk of Bias

Study Name	Selection Bias	Comparability	Outcome Bias	Final ROB
Jiang, et al. 2019	✓	×	✓	×
Demircan et al. 2020	✓	✓	✓	✓
Mitsunaga et al. 2019	✓	✓	✓	✓
Koksal et al. 2016	✓	×	✓	×
Maftoohian et al. 2020	✓	✓	✓	✓
Yuan et al. 2018	✓	✓	✓	✓
Dundar et al. 2016	✓	✓	✓	✓
Graham et al. 2020	✓	✓	✓	✓
Pirneskoski et al. 2019	✓	✓	✓	✓
Martin-Rodriguez et al. 2019a	✓	✓	✓	✓
Martin-Rodriguez et al. 2020a	✓	✓	✓	✓
Martin-Rodriguez et al. 2019b	✓	✓	✓	✓
Vihonen et al. 2020	✓	✓	✓	✓
Magnusson et al. 2020	✓	✓	✓	✓
Martín-Rodríguez et al. 2020b	✓	✓	✓	✓
Geier,2013	✓	✓	✓	✓
van der Woude,2018	✓	✓	✓	✓
Vorwerk,2008	✓	✓	✓	✓
Saeed,2019	✓	✓	✓	✓
Brink,2019	✓	✓	✓	✓
