# Supplementary material for: The use of early warning system scores in prehospital and emergency department settings to predict clinical deterioration: A systematic review and meta-analysis
Source: PLoS One. 2022 Mar 17;17(3):e0265559. doi: 10.1371/journal.pone.0265559 (PMC8929648; doi:10.1371/journal.pone.0265559)
Supplement: Attachment — Submitted filename: Response to reviewers comments_CRIC-D-21-01286.docx [file pone.0265559.s005.docx]

08/11/2021

**Dear Editor in Chief**

**Re: CRIC-D-21-01286– *“The use of Early Warning System scores in pre-hospital and emergency department settings to predict clinical deterioration: a systematic review and meta-analysis”***

*Thank you for the opportunity to revise the manuscript for transferring to other journals. In line with the editor’s and reviewers’ comments, as requested, we have responded on a point-by-point basis and have changed the manuscript accordingly.*

**Reviewer #1**:

Predict future in-hospital clinical worsening from emergency department (ED) or pre-hospital setting with pre-defined scores (as NEWS, NEWS2, MEWS, RAPS and CART). Compare those 5 scores to stated the best one.
Assist the prediction of a possible in-hospital deterioration - see as ICU admission or mortality from 3 to 30 days- from the emergency department (or pre-hospital setting) with scores. Five scores were competed: NEWS, NEWS2, MEWS, RAPS and CART.
EWS scores' predictability of clinical deterioration improves when applied on patient populations that are already in the ED or hospital. The high thresholds used and the scores' failure to predict 30-day mortality make them less suited for use in the pre-hospital setting

Major comments

Comment 1
It appears that the elimination of a very impressive number of articles was necessary to be able to build a meta-analysis on only 15 articles (8 hospital emergency and 7 pre-hospital)

*Authors’ answer:*

***This systematic review followed specific inclusion and exclusion criteria. All studies that did not meet our inclusion criteria were excluded. The objective of this systematic review was to compare the EWS cut-off points in predicting patient outcomes. Therefore, only studies that reported cut-off points were included. It is very commonplace that in systematic reviews and meta-analyses, a limited number of studies are usually included depending on the utilized inclusion and exclusion criteria and this was the case in our study.***

Comment2:

We can also notice that articles in the emergency department more often concern patients over 60 years old. This is not the case for pre hospital articles. Age is not a criterion in evaluation scores. Do you think this should be taken into account?

*Authors’ answer:*

***This systematic review included patients aged ≥14 years. Age was not regarded as a criterion in evaluating the different scores. However, in the meta-regressions, we investigated whether age was a contributing factor for between study heterogeneity. As stated in the manuscript, age was the only study known factor that contributed to heterogeneity, explaining 92% of the between-study variance in the emergency department papers. In the pre-hospital setting, meta-regression could not be performed due to the limited number of studies.***

Comment 3:

The problem of the choice of thresholds for a chosen index is not sufficiently precise and its apparently insignificance is not enough discussed. You seem to think this problem is secondary, but readers need to better understand why. The major interest in the initial management is to determine the deterioration in the short term (3 days). A longer-term prediction appears less interesting. This does not seem to be your opinion but you do not develop this point. In the emergency room, in most acute situations (trauma, sepsis), there is a much greater interest in predicting an outcome at 5 or 7 days rather than at 30 days. The search for a prognosis of the first week is easily understood in order to guide the future in the hospital and therefore, to decide the place of hospitalisation (ICU or not). The interest of the prognosis at 30 days seems premature in those acute situations. Many other factors can intervene in the long-term outcome such as social conditions, the patient's entourage, age or nosocomial complications. Presence, funding and organisation of the means of care, rehabilitation or social assistance are too important to obtain an exact reflection of the patient's future upon arrival. Or, we must accept as a corollary that long-term care itself and its organisation do not have the capacity to influence the future of a patient once the acute phase has passed. This might sound like a somewhat partisan view of medicine.
I'm having trouble understanding the interest of predicting a 30-day outcome from an acute problem that warrants an emergency medical consultation. It seems rather reassuring not to be able to easily achieve this. It is therefore somewhat reassuring that the prediction is weaker to become so in the long term.

*Authors answer:*

***We thank the reviewer for this thoughtful comment. Based on the retrieved and other known literature, clinicians use EWS scores to predict both short term and long-term outcomes. This is consistently reported. Currently there are no guidelines to whether these scores predict well long-term outcomes. This is the first systematic review and meta-analysis that answers this question.***

***Understandably, short-term mortality is of relevance and of concern following a clinical deterioration; however, long term outcomes such as 30-day mortality are also investigated and reported in the literature.***

***We agree with the reviewer’s comments that the prediction of 30 days or long-term mortality may be subject to many confounding factors, such as comorbidities and management care provided in the pre-hospital and hospital settings. Subsequent clinical deteriorations may occur following admission and these may impact long-term outcomes like 30-day mortality. We have added a limitation relating to this point.***

***In this systematic review, we reported both short-term and long-terms outcomes as these are reported as outcomes of interest and are continuously used by clinicians. This systemic review aims to provide clinical evidence to clinicians that different cut-off points may have similar predictability and that the prediction of immediate clinical deterioration (i.e., 3-day mortality) is better achieved by these scores as compared to longer term outcomes such as 30-day mortality although we did find that these scores do predict also long-term mortality when patients are already in the hospital setting.***

Comment 4:

Otherwise, it also seems obvious that the prediction is less strong in the hospital pre-hospital than in the emergency room. There are many possibilities for this difference: evolution of the disease and the delay between pre-hospital and hospital examination, type of caregiver doing the score and its experience, perhaps also easier examinations in hospital.

*Authors' answer:*

***Using evidence-based research methods, this systematic review compared the pre-hospital and ED settings. In the literature there is no evidence on whether the scores’ predictability would differ by the setting. This study provides the clinicians, both in the ED and pre-hospital settings, valid tools to consider when predicting patient outcomes. In the pre-hospital setting studies, the scores were administered by researchers utilizing the parameters in the pre-hospital setting, thus type of caregiver or their experience in using this score is less relevant to our systematic review results.***

Comment 5

Moreover, what is the place of doctors in prediction measurements? Pre-hospital care in many countries is provided by paramedics (like Australia if I'm not mistaken). You never discuss a possible difference between a medical diagnostic and triage in the ED or pre hospital (or even the possibility to evaluate and choice in the first step when people phone to have help) and a paramedical scoring.

*Authors answer:*

***As stated earlier, the scores used in the pre-hospital setting were applied by the researchers conducting the studies. The researchers used the parameters recorded for patients treated in the pre-hospital setting, namely the vital signs of the patients. This study did not rely on diagnoses made in either the pre-hospital or ED setting but rather on the actual vital signs of the patients. There is no evidence to indicate that vital signs measured by physicians and paramedics will differ based on the profession of the person measuring them.***

Comment 6
In total, the article is not precise enough on the interest of the scores measured in acute but also on the organisation of pre-hospital care. The differences in efficiency of the scores found in the emergency department compared to the pre-hospital are not sufficiently analysed. The article emphasises the lack of precision of these long-term scores but the relevance of these in this context seems questionable

*Authors answer:*

***We disagree with the reviewer. Using published literature our review aimed to inform about the predictability of the different EWS scores being used in clinical practice in either the ED or pre-hospital setting. In systematic reviews it is impossible to account for the variability between these two settings mostly because we do not have the actual data from each of the included studies and there are many confounders that have not been accounted for in all studies. The aim of all systematic reviews including this one is to use available literature and to summarise the data based on available literature. Despite the less than satisfactory long term mortality predicting ability of EWS scores, they still provide a valued evidence-based tool for clinicians, in the ED or pre-hospital setting alike.***

**Reviewer #2**

The article by Guan and colleagues presents a meta-analysis evaluating the predictive ability of five commonly used early warning scores in dependence whether they have been applied in the pre-hospital setting or the emergency department.

The topic of the paper falls within the scope of Critical Care. The background, methods, and results are well-described, and the conclusions are fair based on the data analysed. The language quality is high. The findings are novel, but somewhat unimpressive. Whether the paper is of sufficient interest for publication is an editorial decision.

Comment 1:
There are some minor issues that can be easily addressed:
1) Keywords: Why capitalise prediction but not mortality?

Authors answer:

**This has been addressed in the updated manuscript. Both are now capitalised.**

Comment 2:
2) In limitations, please consider taking the following articles or the issues presented similar articles into account https://www.resuscitationjournal.com/article/S0300-9572(15)00076-3/fulltext
https://academic.oup.com/jamiaopen/article/2/4/465/5555857 (the reviewer has no conflict of interest for both)

*Authors answer:*

**Thank you. We have cited one of the suggested papers. We agree that miscalculation of such scores may always occur as human error is always possible in all tools that are completed by humans. Automation of such systems can reduce the probability of human error although human error can always occur even in recording the vital signs. This systematic review does not attempt to develop or relate to a "new score". This review aimed to compare different EWS cut-points aiming to provide evidence-based research that can be used by clinicians in decision making regarding the choice of the best EWS score in both the ED and pre-hospital settings.**

Comment 3:
3) Page 5 line 15 the line breaks seem odd.

Authors answer:

**Link breaks have been removed.**

Comment 4:

4) In limitations, please consider discussing that the ED happens "after" the pre-hospital setting, therefore, a certain amount of treatment may have already occurred affecting the results.

Authors answer:

**We have addressed this in the revised manuscript.**

Comment 6:
5) Page 5 Line 45 and onwards. The reviewer is somewhat puzzled that patients with Sepsis were excluded. This should be stated in the limitations, since these patients especially benefit for being properly assessed early on.

Authors answer:

**As suggested by the reviewer, in the revised manuscript we have included sepsis papers that met the study inclusion criteria. Since often deranged scores are observed in sepsis patients, their analysis was conducted separately as shown in the revised forest plot. Adding sepsis patients did not change the main conclusions of this systematic review.**

Yours sincerely,

Gigi Guan

La Trobe Rural Health School

College of Science, Health and Engineering | La Trobe University | Victorial, Australia
Email: g.guan@latrobe.edu.au
