# Supplementary material for: The use of early warning system scores in prehospital and emergency department settings to predict clinical deterioration: A systematic review and meta-analysis
Source: PLoS One. 2022 Mar 17;17(3):e0265559. doi: 10.1371/journal.pone.0265559 (PMC8929648; doi:10.1371/journal.pone.0265559)
Supplement: Attachment — Submitted filename: Response to reviewers comments_PONE-D-21-35942.docx [file pone.0265559.s006.docx]

22/01/2022

Dear Dr Yong-Hong Kuo

Academic Editor

*PloS One*

**Re: PONE-D-21-35942– “*The Use of Early Warning System Scores in Prehospital and Emergency Department Settings to Predict Clinical Deterioration: A Systematic Review and Meta-Analysis”***

Thank you for the reviewers’ consideration and kind words regarding this systemic review and meta-analysis. In line with the editor’s and reviewers’ comments, as requested, we have responded on a point-by-point basis and have changed the manuscript accordingly.

**Reviewer #1**


Comment 1:

This is the well compared study showing differences in hospital setting and pre hospital setting.

***Authors’ response:***

***We thank the reviewer for these positive comments.***

Comment 2:

We also use qSOFA for pre hospital and SOFA for emergency. As the study has used sepsis case and we are using SOFA or qSOFA to predict the outcome.Using this predictors also in the early warning score would have made more clear for the reader. As pre hospital setting has been used for patient treated by medical care personnel in this study .Cases from those category also referred when they become critical.And we usually understand the management in EMS while transporting patient to the definitive care center. So it would be good for reader if this has been clear in the initial part about pre hospital.

***Authors’ response:***

***We acknowledge the reviewer’s comment.***

***The aim of this SR was to investigate the performance of the five EWS scores, in predicting clinical deterioration, that are most commonly used in both the pre hospital and emergency department (ED) settings. Although, as the reviewer has correctly stated, both SOFA or qSOFA are used to predict clinical deterioration in patients experiencing sepsis, these scores are not commonly used in both the pre hospital and ED settings. That was the reason why these were not initially included in this SR. The aim of this SR was to limit the scores to those that do not require advanced clinical testing; thus, SOFA was not considered.***

**Reviewer # 2**

Comment

The authors have in their revised version adequately addressed the strengths and limitations in the submitted paper. Although the application of EWS in prehospital settings has many drawbacks and is problematic the authors have clearly stated the choice of thresholds, cut-off-points in different settings: The statement in the conclusion that EWS scores applied in the prehospital setting cannot accurately predict long-term events but can predict short term clinical decline puts the results of the systematic review/metaanalyses in the right perspective.

***Authors’ response:***

***We thank the reviewer for these positive comments.***

**As suggested by the editor, we have updated the following reference:**

**We have identified a reference duplication has occurred, F. Martin-Rodriguez, R. Lopez-Izquierdo, C. Del Pozo Vegas, J. F. Delgado Benito, P. Del Brio Ibanez, I. Moro Mangas, et al. This now has been rectified.**

**We have also updated the reference when citing the Newcastle-Ottawa Scale for risk of bias assessment. The updated reference has been added.**

We thank you the reviewers and editor for the comments, and appreciate the time invested in reviewing this article.

Yours sincerely,

Gigi Guan

La Trobe Rural Health School

College of Science, Health and Engineering | La Trobe University | Victoria, Australia
Email: g.guan@latrobe.edu.au
